# Molecular Characterization and Comparative Genomics of Two *Staphylococcus pseudintermedius* Strains from Humans in Egypt

**DOI:** 10.3390/vetsci13050424

**Published:** 2026-04-27

**Authors:** Ola K. Elsakhawy, Haitham Elaadli, Yassien Badr, May Raouf, Stephen A. Kania, Hend Altaib, Mohamed A. Abouelkhair

**Affiliations:** 1 Department of Veterinary Biomedical Sciences, Rowan University, Glassboro, NJ 08062, USA; 2 Department of Animal Hygiene and Zoonoses, Faculty of Veterinary Medicine, Alexandria University, Alexandria 22758, Egypt; 3 Department of Infectious Diseases and Epidemics, Faculty of Veterinary Medicine, Damanhour University, Damanhour 22511, Egypt; 4 Department of Medical Microbiology and Immunology, Faculty of Medicine, Alexandria University, Alexandria 21131, Egypt; 5 Department of Biomedical and Diagnostic Sciences, University of Tennessee, Knoxville, TN 37996, USA; 6 Research and Development Department, Middle East for Vaccines (MEVAC), El Sharqia 44813, Egypt

**Keywords:** Genomics, Egypt, *Staphylococcus pseudintermedius*, MLST, Pangenome, Comparative Genomics

## Abstract

*Staphylococcus pseudintermedius* is an opportunistic bacterium previously associated with dogs but has recently been found in human infections, raising zoonotic concerns. Genomic characterization of human *S. pseudintermedius* isolates can provide preliminary information on antibiotic resistance, pathogenicity, and genomic features relevant to host range. Two *S. pseudintermedius* isolates (hereafter referred to as *S. pseudintermedius* EGH1 and *S. pseudintermedius* EGH2) from human clinical samples in Egypt were sequenced using the Illumina NovaSeq X Plus platform. To assess genetic relatedness to human *S. pseudintermedius* isolates worldwide, multilocus sequence typing (MLST), pangenome analysis, and antimicrobial resistance gene profiling were performed. The sequencing produced a total of 9,499,989 reads for *S. pseudintermedius* EGH1 and 9,567,531 reads for *S. pseudintermedius* EGH2. Sequences were assembled with Geneious Prime^®^ 2025 and annotated using NCBI Prokaryotic Genome Annotation Pipeline v6.10. Pangenome analysis identified 9574 genes, comprising 1681 core genes (17.56%), 180 soft-core genes (1.88%), 837 shell genes (8.74%), and 6876 cloud genes (71.82%). MLST was conducted on human *S. pseudintermedius* genome assemblies using MLST v2.23.0. The analysis revealed both isolates as novel sequence types: *S. pseudintermedius* EGH1 was assigned ST-3037 with a new allele (*purA*-107), and *S. pseudintermedius* EGH2 was assigned ST-2874. Clonal relationships among *S. pseudintermedius* isolates were evaluated using the eBURST algorithm. This study presents the first next-generation genome sequencing and comparative genomic analysis of *S. pseudintermedius* isolates from humans in Egypt. Future studies integrating genomic, epidemiological, and phenotypic data are required.

## Simple Summary

*Staphylococcus pseudintermedius* is a bacterial species commonly found in dogs, but has recently been detected in human infections, raising zoonotic concerns. In our study, we isolated two strains of *Staphylococcus pseudintermedius* from clinical samples obtained from human patients in Egypt and sequenced their complete genomes for the first time. We then compared these samples with those from different countries to explore their relationships and identify genes that may confer antibiotic resistance or enhance pathogenicity. The findings highlight the need for expanded genomic surveillance of *S. pseudintermedius* in North Africa and at the animal–human interface. Future studies should integrate genomic, epidemiological, and phenotypic data for a comprehensive understanding.

## 1. Introduction

Species of the *Staphylococcus* genus are commensal bacteria that live on the skin surface and the mucosa of the upper respiratory tract of animals and humans; however, they can cause opportunistic infections in both hosts [1,2]. *S. pseudintermedius* can cause a variety of infections in companion animals, including pyoderma, otitis externa, and post-surgical wound infections [3].

The prevalence of human colonization by *S. pseudintermedius* is unknown, as it can often be misidentified as *Staphylococcus aureus* [4,5,6]. A study found nasal colonization in 4.1% of humans with pets compared to 27.7% for *S. aureus*, and a lack of handwashing after handling pets was significantly associated with this colonization [7]. Additionally, 3.9% of small-animal dermatologists were colonized with MRSP [8]. Owners of dogs with pyoderma were more likely to test positive for *S. pseudintermedius* [9].

A key challenge in treating infections caused by *S. pseudintermedius* is its methicillin (β-lactam) resistance, caused by the penicillin-binding protein 2a (PBP2a) encoded by the *mecA* gene [10]. Since the first phenotypic characterization of Methicillin-resistant *Staphylococcus pseudintermedius* (MRSP) in the 1980s, MRSP prevalence has surged, rising from below 5% in 2001 to about 30% in 2008 at one U.S. veterinary center. The prevalence of MRSP in humans remains largely unknown, primarily due to frequent misidentification as *S. aureus*. Furthermore, the established breakpoints for *mecA*-mediated resistance are not applicable to *S. pseudintermedius*, leading to inaccurate susceptibility test results [6].

Comparative genomic analyses have provided valuable insights into the genetic makeup, virulence, and antimicrobial resistance mechanisms of *S. pseudintermedius* [11,12,13,14]. Whole-genome sequencing (WGS) has revealed a high level of genetic diversity within the species, with distinct clonal lineages exhibiting different host-adaptation and antimicrobial-resistance profiles [15,16]. Despite these advances, information on the genomic characteristics of *S. pseudintermedius* isolates from Egypt remains limited, especially regarding strains isolated from human hosts. Regional differences in the distribution of sequence types (STs) suggest that local factors, including antibiotic usage patterns, pet ownership trends, and veterinary infection control practices, play a role in the emergence of new or regionally restricted lineages [17]. Understanding the genomic landscape of *S. pseudintermedius* in underrepresented regions, such as North Africa, is therefore essential for clarifying global transmission dynamics and potential zoonotic exchange pathways between companion animals and humans. Comparative genomics offers a powerful approach for identifying and analyzing the genetic factors underlying host adaptation, antimicrobial resistance, and virulence. Pangenome analysis, which separates the core and accessory genomes, provides a framework for examining the evolutionary forces that shape bacterial populations [11]. The accessory genome often contains genes acquired through horizontal gene transfer that give bacteria adaptive benefits under selective pressures, such as antibiotic exposure or host immune responses. In this study, we performed whole-genome sequencing and comparative genomic analysis on two *S. pseudintermedius* strains (hereafter called *S. pseudintermedius* EGH1 and *S. pseudintermedius* EGH2) isolated from human clinical samples in Egypt. Using a combination of multilocus sequence typing (MLST), pangenome analysis (Roary), and antimicrobial resistance gene profiling (AMRFinderPlus), we assessed the genetic relationship of these isolates compared to a global collection of human *S. pseudintermedius* genomes. Our objectives were to (i) describe the genomic features of these two Egyptian human isolates in the context of global lineages and (ii) examine the distribution of antimicrobial resistance genes. Although limited to two isolates, this study provides initial genomic data from an underrepresented region and underscores the need for broader surveillance at the interface between veterinary and human infections.

## 2. Materials and Methods

### 2.1. Bacterial Strains, Media, and Growth Conditions

A cross-sectional study was conducted from March 2022 to November 2022 in the Alexandria governorate of Egypt. During this period, a total of 174 pus swabs were collected from septic wounds of human patients at Alexandria University Hospital. Bacteria were propagated in this study as previously described [18]. Samples were individually inoculated by streaking on defibrinated 5% sheep blood agar and incubated aerobically overnight at 37 °C to identify beta-hemolytic activity, following laboratory procedures at the Faculty of Medicine, Alexandria University, Egypt. Catalase-positive, Gram-positive cocci were subcultured directly into mannitol salt agar for selective isolation of Staphylococci.

### 2.2. DNA Extraction, Library Preparation, and Whole-Genome Sequencing

DNA was extracted as previously described and purified using the MagMAX Viral/Pathogen Nucleic Acid Isolation Kit (Thermo Fisher Scientific, Waltham, MA, USA) [18]. The DNA quantity and quality were evaluated using a NanoDrop 2000 (Thermo Fisher Scientific, USA) and Qubit fluorometer (Fisher, Waltham, MA, USA). Illumina sequencing libraries and sequencing were performed by SeqCenter in Pittsburgh, PA, USA. Illumina sequencing libraries were prepared using the tagmentation-based and PCR-based Illumina DNA Prep kit (Illumina, San Diego, CA, USA) and custom IDT 10 bp unique dual indices (UDI) with a target insert size of 280 bp (Integrated DNA Technologies, Coralville, IA, USA). No additional DNA fragmentation or size selection steps were performed. Illumina sequencing was performed on an Illumina NovaSeq X Plus sequencer (SeqCenter in Pittsburgh, PA) in a single multiplexed, shared-flow-cell run, producing 2 × 151 bp paired-end reads. Demultiplexing, quality control, and adapter trimming were performed with bcl-convert (v4.2.4). Sequences were de novo assembled using Geneious Prime^®^ 2025 [19]. The quality of the assembled genomes was determined using the quality assessment tool for genomic assemblies (QUAST v5.3.0) [20]. The annotation of each isolate was performed using the NCBI Prokaryotic Genome Annotation Pipeline v6.10.

### 2.3. Multi-Locus Sequence Typing

Multi-locus sequence typing (MLST) was performed on *S. pseudintermedius* EGH1 and *S. pseudintermedius* EGH2 genome assemblies using MLST v2.23.0 (https://github.com/tseemann/mlst, accessed on 1 October 2025) within a Docker container (staphb/mlst:latest, platform linux/amd64) [21]. The analysis employed the *S. pseudintermedius* MLST scheme, which comprises seven housekeeping genes: *ack*, *cpn60*, *fdh*, *pta*, *purA*, *sar*, and *tuf*. Allelic profiles generated by MLST were matched against the complete PubMLST *S. pseudintermedius* sequence definitions database (accessed 1 October 2025, containing 3037 sequence types) to assign sequence types (STs) to each isolate. The database was exported from the PubMLST platform (https://pubmlst.org/organisms/staphylococcus-pseudintermedius, accessed on 1 October 2025) and contained all allelic profiles and ST designations available at the time of analysis [22]. Profile matching was performed using exact allele–number comparisons across all seven loci, with isolates receiving ST assignments only when their complete seven-locus profiles matched existing database entries.

We queried the publicly accessible BIGSdb database (https://pubmlst.org/bigsdb?db=pubmlst_spseudintermedius_isolates&l=1&page=query, accessed on 12 October 2025) for *S. pseudintermedius* human isolates and retrieved all matching records [22].

Clonal relationships among typed isolates were analyzed using the eBURST (Based Upon Related Sequence Types) algorithm (https://www.mlst.net/eburst/, accessed on 15 October 2025).

### 2.4. Pangenome Analysis

Genome sequences of *S. pseudintermedius* were retrieved from the NCBI Pathogen Detection database (https://www.ncbi.nlm.nih.gov/pathogens, accessed on 16 October 2025) using the search term “*Staphylococcus pseudintermedius*”. The search results were filtered by organism group and host to include only isolates associated with *Homo sapiens*. A total of 310 publicly available human-source *S. pseudintermedius* genome assemblies were retrieved and used as the comparative dataset Table S1. Then, genome assemblies were downloaded using the “dehydrated download” feature available through the NCBI Datasets tool (https://www.ncbi.nlm.nih.gov/pathogens/docs/datasets_assemblies/). The downloaded datasets were subsequently rehydrated and extracted to FASTA format for downstream comparative genomic and phylogenetic analyses.

Genome assemblies were annotated using Prokka v1.14.5 annotation (https://github.com/tseemann/prokka) with default parameters to generate GFF3 files for downstream analysis [23]. The annotated files were then analyzed using the Roary v3.13.0 pangenome pipeline (https://github.com/sanger-pathogens/Roary) with default settings [24]. The output included gene classification into core, soft-core, shell, and cloud categories based on their frequency across all genomes. The pangenome matrix and core gene alignment generated by Roary were subsequently used for phylogenetic reconstruction and visualization.

### 2.5. Antimicrobial Resistance Genes

Antimicrobial resistance genes were identified in *S. pseudintermedius* EGH1 and EGH2 using AMRFinderPlus v4.0.23 in combined nucleotide and protein modes (https://github.com/ncbi/amr/releases) [25].

## 3. Results

### 3.1. Genomic Features of S. pseudintermedius EGH1 and EGH2

*S. pseudintermedius* EGH1 and *S. pseudintermedius* EGH2 were sequenced on the NovaSeq X Plus platform, producing 2 × 151 bp paired-end reads. Sequences were assembled with Geneious Prime^®^ 2025 and annotated using NCBI Prokaryotic Genome Annotation Pipeline v6.10.

The number of reads, genome length, N50 values, number of contigs, GC percent, genome coverage, and number of genes of the genome sequences are listed in Table 1.

**Figure 1:**
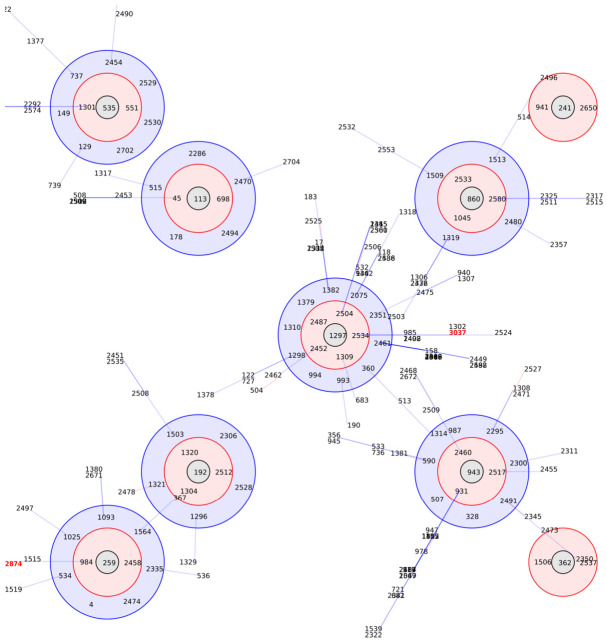
Analysis of clonal complexes of human *Staphylococcus pseudintermedius* isolates using the eBURST algorithm. Single locus variant (SLV) profiles that match the central profile are indicated within a red circle, while double locus variant (DLV) profiles are marked within a blue circle. More distant profiles, referred to as triple locus variants, are connected by a line. Sequence types of *S. pseudintermedius* EGH1 (ST 3037) and EGH2 (ST 2874) are highlighted in red.

MLST was conducted on human *S. pseudintermedius* genome assemblies using MLST v2.23.0. The analysis identified both isolates as novel sequence types: *S. pseudintermedius* EGH1 was assigned ST-3037, which carries a new allele (*purA*-107), while *S. pseudintermedius* EGH2 was assigned ST-2874 Table 2. Population structure and clonal relationships among typed isolates were examined using the eBURST (Based Upon Related Sequence Types) algorithm. Single-locus variants (SLVs), defined as isolates differing at exactly one of the seven MLST loci, were identified to construct a minimum spanning tree of clonal relationships. Clonal complexes (CCs) were defined as groups of three or more related STs connected through SLV relationships, with the most common ST designated as the founder of each complex Figure 1.

### 3.2. Pangenome Analysis

**Figure 2:**
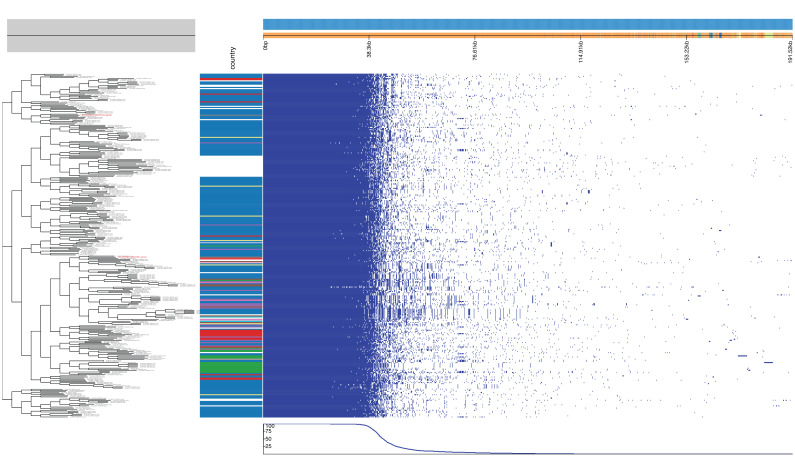
Pangenome analysis of human *S. pseudintermedius* genomes using Roary. The left panel shows a Bayesian phylogenetic tree constructed from single-nucleotide polymorphisms (SNPs) in the core genome. The right panel presents a Roary gene presence/absence matrix, where each column represents an isolate and each row corresponds to a gene cluster. Blue shading indicates the presence of a gene, while white denotes its absence. Isolates from different countries are color-coded as follows: USA (#1f77b4), Trinidad and Tobago (#ff7f0e), Brazil (#2ca02c), United Kingdom (#d62728), South Africa (#9467bd), Japan (#8c564b), Thailand (#e377c2), Egypt (#7f7f7f), Australia (#bcbd22), Switzerland (#17becf), Ireland (#aec7e8), New Zealand (#ffbb78), France (#98df8a), Hungary (#ff9896), South Korea (#c5b0d5), Argentina (#c49c94), Germany (#f7b6d2), Sweden (#c7c7c7), not collected (#dbdb8d).

**Figure 3:**
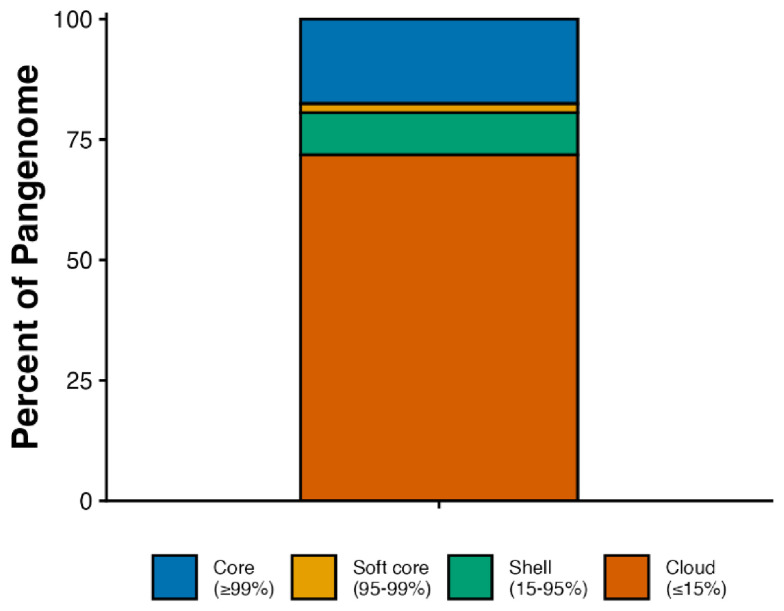
*Pangenome composition of human Staphylococcus pseudintermedius isolates analyzed using Roary.* The figure illustrates the proportion of genes classified as core (present in all genomes, blue: #0072B2), soft-core (present in 95–99% of genomes, orange: #E69F00), shell (present in 15–95% of genomes, green: #009E73), and cloud (present in <15% of genomes, red#D55E00).

A total of 310 publicly available human-source *S. pseudintermedius* genome assemblies were retrieved from the NCBI Pathogen Detection database (accessed 16 October 2025) and used as the comparative dataset Table S1. These isolates originated from different countries, with the majority from the USA, and were associated with diverse clinical presentations; see Figure 2: Pangenome analysis of human *S. pseudintermedius* genomes using Roary. Pangenome analysis revealed a total of 9574 genes, comprising 1681 core (17.56%), 180 soft-core (1.88%), 837 shell (8.74%), and 6876 cloud genes (71.82%); see Figure 3. Eighty-five genes were found to be unique to the Egyptian isolates, with a significant number identified as hypothetical proteins, alongside the putative ATP-dependent DNA helicase yoaA.

A total of 85 genes were identified as unique to both isolates, including those coding for the putative ATP-dependent DNA helicase yoaA, several metabolic and cell-surface-associated proteins, and 62 hypothetical proteins. Conversely, 6890 genes found in other isolates were absent from EGH1 and EGH2. Additionally, 2599 genes were shared between the two isolates and at least some of the other genomes.

### 3.3. Antimicrobial Resistance Genes

AMRFinderPlus analysis identified multiple antimicrobial resistance (AMR) genes in both isolates. In *S. pseudintermedius* EGH1, genes conferring aminoglycoside resistance (*ant(6)-Ia*, *aph(3′)-IIIa*), β-lactam resistance (*blaI*, *blaPC1*, *blaR1*), chloramphenicol resistance (*catA*), macrolide-lincosamide-streptogramin B resistance (*erm(B)*), fusidic acid resistance (*fusC*), streptothricin resistance (*sat4*), and tetracycline resistance (*tet(M)*) were detected Table 3.

In *S. pseudintermedius* EGH2, genes associated with β-lactam resistance (*blaI*, *blaPC1*, *blaR1*), trimethoprim resistance (*dfrG*), fusidic acid resistance (*fusC*), and tetracycline resistance (*tet(K)*) were identified and are shown in Table 3.

## 4. Discussion

*S. pseudintermedius* is a coagulase-positive bacterium frequently found as a commensal organism on the skin and mucous membranes of dogs and, less often, cats. However, in recent years, *S. pseudintermedius* has emerged as an opportunistic pathogen responsible for a wide range of infections in companion animals. Its increasing detection in humans, especially among pet owners, veterinarians, and immunocompromised individuals, underscores its growing significance as a zoonotic agent of clinical importance [26,27,28].

Whole-genome sequencing (WGS) and comparative genomics have revealed a high level of genetic diversity within the species, with distinct clonal lineages exhibiting different host-adaptation and antimicrobial-resistance profiles [15,16]. Despite these advances, information on the genomic characteristics of *S. pseudintermedius* isolates from Egypt remains limited, especially regarding strains isolated from human hosts. Understanding the genomic landscape of *S. pseudintermedius* in underrepresented regions, such as North Africa, is therefore essential for clarifying global transmission dynamics and potential zoonotic exchange pathways between companion animals and humans. In this study, we performed whole-genome sequencing of two human *S. pseudintermedius* isolates from Egypt. Using a combination of multilocus sequence typing (MLST), pangenome analysis (Roary), and antimicrobial resistance gene profiling (AMRFinderPlus), we assessed the genetic relationship of these isolates to a global collection of human *S. pseudintermedius* genomes. Although limited to two isolates, this study provides initial genomic data from an underrepresented region and underscores the need for broader surveillance at the interface between veterinary and human infections.

AMRFinderPlus analysis revealed the presence of AMR genes in both isolates. In *S. pseudintermedius* EGH1, genes associated with resistance to aminoglycosides, β-lactams, chloramphenicol, macrolide-lincosamide-streptogramin B, fusidic acid, streptothricin, and tetracycline were detected. Meanwhile, *S. pseudintermedius* EGH2 harbored genes conferring resistance to β-lactams, trimethoprim, fusidic acid, and tetracycline. These findings, consistent with previous studies, highlight the urgent need for robust strategies to tackle AMR and for integrated One Health surveillance to curb its spread at the animal–human interface [29,30,31].

The pangenome analysis revealed a highly dynamic accessory genome, with 9574 total gene clusters spread across a relatively small core genome (1681 genes, 17.56%) and a large cloud genome (6876 genes, 71.82%). Both EGH1 and EGH2 shared core genomic features typical of *S. pseudintermedius*. This pangenome structure, characterized by a large accessory gene pool relative to the conserved core, has been observed in bacterial species occupying diverse niches and undergoing frequent horizontal gene transfer [32]. It should be noted, however, that including only two Egyptian isolates limits the conclusions that can be drawn about regional genomic diversity, and the patterns described here should be considered preliminary.

Among the 85 genes identified as uniquely present in EGH1 and EGH2 were genes involved in capsule biosynthesis (*capC*), teichoic acid biosynthesis (*tarL* and *tarJ*), and peptidoglycan synthesis. In other staphylococcal species, variation in teichoic acid structure has been associated with altered immune recognition and reduced antimicrobial susceptibility [33,34]. Whether these genes confer a similar functional advantage in the two Egyptian isolates remains to be determined through phenotypic and larger-scale comparative studies.

Several limitations in this study warrant consideration. Most notably, this study is based solely on two human isolates of *S. pseudintermedius* from Egypt. As a result, all findings regarding genomic content, resistance profiles, and host-associated characteristics should be considered preliminary and cannot be generalized to the broader *S. pseudintermedius* population in Egypt or North Africa. Furthermore, the comparative dataset is predominantly composed of isolates from the USA, which may introduce bias into population-structure analyses and restrict the applicability of the findings to global populations. Clinical metadata—including infection type, patient risk factors, and treatment outcomes—were not available for the isolates examined in this study, precluding genotype–phenotype associations. Future studies that collect standardized clinical data alongside whole-genome sequencing would enable such comparisons.

## 5. Conclusions

This study presents the first next-generation genome sequencing and comparative genomic analysis of *S. pseudintermedius* isolates from humans in Egypt. *S. pseudintermedius* EGH1 and EGH2 harbor unique genetic elements, multiple hypothetical proteins, and a diverse profile of antimicrobial resistance genes. Expanding genomic surveillance in North Africa and beyond will be important for building a more representative picture of *S. pseudintermedius* diversity in this region. Future studies with larger sample sizes, integrating genomic, epidemiological, and phenotypic data, will be needed to clarify the clinical significance and population dynamics of *S. pseudintermedius* across human and veterinary settings.

## Figures and Tables

**Table 1:** Characteristics of the genome sequences of *S. pseudintermedius* EGH1 and *S. pseudintermedius* EGH2.

	EGH1	EGH2
Number of reads	9,499,989	9,567,531
Genome length	2,536,812 bp	2,636,008 bp
N50	141,100 bp	139,200 bp
Number of contigs	41	99
GC percent	37.5%	37.5%
Genome coverage	9.22×	9.29×
Genes	2424	2596

**Table 2:** MLST profile of the two human *S. pseudintermedius* isolates.

Isolate	*ack*	*cpn60*	*fdh*	*pta*	*pura*	*Sar*	*tuf*	Sequence Type (ST)
*S. pseudintermdius* EGH1	6	2	1	1	107	1	2	3037
*S. pseudintermdius* EGH2	1	18	2	2	3	1	1	2874

**Table 3:** Antimicrobial resistance genes identified in *S. pseudintermedius* EGH1 and EGH2.

Antimicrobial Class	Gene	EGH1	EGH2
**Aminoglycoside**	*ant(6)-Ia*	+	−
	*aph(3′)-IIIa*	+	−
**β-Lactam**	*blaI*	+	+
	*blaPC1*	+	+
	*blaR1*	+	+
**Chloramphenicol**	*catA*	+	−
**Fusidic acid**	*fusC*	+	+
**Macrolide–lincosamide–streptogramin B**	*erm(B)*	+	−
**Streptothricin**	*sat4*	+	−
**Tetracycline**	*tet(M)*	+	−
	*tet(K)*	−	+
**Trimethoprim**	*dfrG*	−	+

## Data Availability

The data presented in this study are openly available in the NCBI BioProject under accession number: PRJNA1285324. *S. pseudintermedius* isolates EGH1 and EGH2 complete genome sequences were deposited in NCBI under accession numbers SRR34336090 and SRR34336089, respectively. Genome assemblies were deposited under accession numbers GCA_051374085.1 and GCA_051374445.1.

## Supplementary Materials

The following supporting information can be downloaded at: https://www.mdpi.com/article/10.3390/vetsci13050424/s1.
